# Deep learning-based artificial intelligence model for classification of vertebral compression fractures: A multicenter diagnostic study

**DOI:** 10.3389/fendo.2023.1025749

**Published:** 2023-03-22

**Authors:** Fan Xu, Yuchao Xiong, Guoxi Ye, Yingying Liang, Wei Guo, Qiuping Deng, Li Wu, Wuyi Jia, Dilang Wu, Song Chen, Zhiping Liang, Xuwen Zeng

**Affiliations:** ^1^ Department of Radiology, Guangzhou Red Cross Hospital (Guangzhou Red Cross Hospital of Jinan University), Guangzhou, China; ^2^ Department of Radiology, Guangzhou First People’s Hospital, Guangzhou, Guangdong, China; ^3^ Department of Radiology, Wuhan Third Hospital, Tongren Hospital of Wuhan University, Wuhan, Hubei, China; ^4^ Department of Radiology, Hubei 672 Integrated Traditional Chinese and Western Medicine Orthopedic Hospital, Wuhan, Hebei, China

**Keywords:** vertebral compression fractures, DL: deep learning, DR: digital radiography, x-ray, CNN

## Abstract

**Objective:**

To develop and validate an artificial intelligence diagnostic system based on X-ray imaging data for diagnosing vertebral compression fractures (VCFs)

**Methods:**

In total, 1904 patients who underwent X-ray at four independent hospitals were retrospectively (n=1847) and prospectively (n=57) enrolled. The participants were separated into a development cohort, a prospective test cohort and three external test cohorts. The proposed model used a transfer learning method based on the ResNet-18 architecture. The diagnostic performance of the model was evaluated using receiver operating characteristic curve (ROC) analysis and validated using a prospective validation set and three external sets. The performance of the model was compared with three degrees of musculoskeletal expertise: expert, competent, and trainee.

**Results:**

The diagnostic accuracy for identifying compression fractures was 0.850 in the testing set, 0.829 in the prospective set, and ranged from 0.757 to 0.832 in the three external validation sets. In the human and deep learning (DL) collaboration dataset, the area under the ROC curves(AUCs) in acute, chronic, and pathological compression fractures were as follows: 0.780, 0.809, 0.734 for the DL model; 0.573, 0.618, 0.541 for the trainee radiologist; 0.701, 0.782, 0.665 for the competent radiologist; 0.707,0.732, 0.667 for the expert radiologist; 0.722, 0.744, 0.610 for the DL and trainee; 0.767, 0.779, 0.729 for the DL and competent; 0.801, 0.825, 0.751 for the DL and expert radiologist.

**Conclusions:**

Our study offers a high-accuracy multi-class deep learning model which could assist community-based hospitals in improving the diagnostic accuracy of VCFs.

## Introduction

Vertebral compression fractures (VCFs) are common diseases that seriously affect human life and pose a very large challenge to the health care system (1). With the rising prevalence of population aging, the occurrence of VCFs due to trauma and osteoporosis is increasing year by year, which increases societal and familial economic burdens. Moreover, pathologic fractures resulting from neoplasms are another leading cause of VCFs worldwide. All types of VCFs foreshadow a high risk of poor outcomes, so early, personalized and effective medical intervention is strongly advised. Therefore, it is desirable to find an accurate and effective method to detect and identify acute, chronic and pathological VCFs.

**Figure 1:**
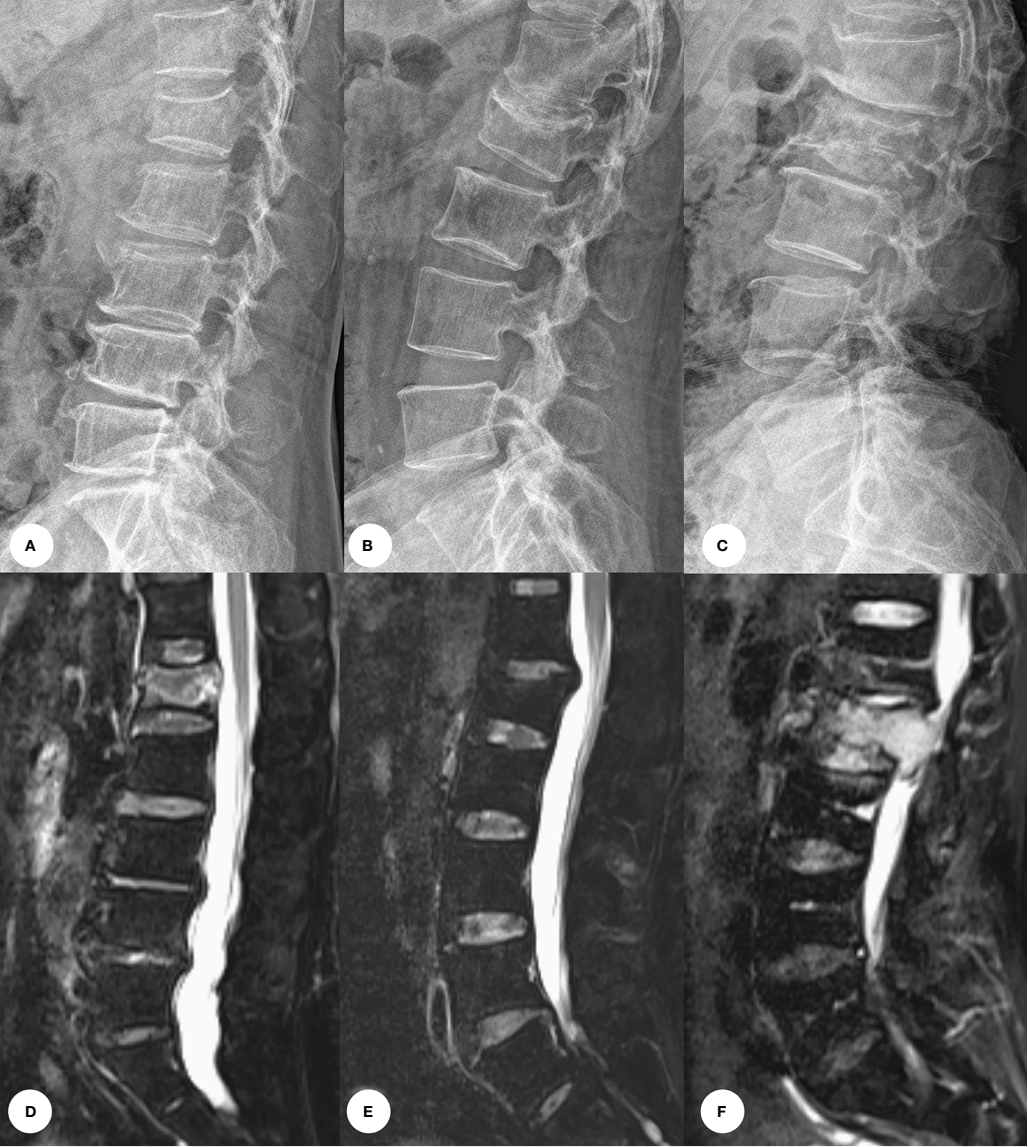
Images of vertebral compression fractures types. **(A, D)** Acute compression fracture of the L1 vertebral; **(B, E)** Chronic compression fracture of the L1 vertebral; **(C, F)** Pathologic compression fracture of the L2 vertebral.

In recent years, the incidence of back pain due to compression fractures has increased in patients. Many imaging methods are available for early screening and differentiation of compression fractures, such as X-ray (XR) images, Computed tomography (CT) and magnetic resonance imaging (MRI). Among these procedures, CT is the modality of choice for the evaluation of bone structure and fragments. MRI may be the most useful imaging technique based on its excellent soft tissue contrast that shows the change in the signal intensity and morphological characteristics of the collapsed vertebrae. Acute compression fractures show hyperintensity with bone marrow edema, while chronic compression fractures show no bone marrow edema and are isointense on T2WI fat-suppression sequences. The pathologic VCFs shows low signal intensity on T1WI, isointensity or high signal intensity on T2WI, and homogeneous or inhomogeneous enhancement (**Figure 1**). However, the availability of CT and MRI is limited for overall population diagnosis due to their complexity, high time consuming and high-cost factor (2). In contrast, X-ray images with effective cost and time may be an attractive and widespread method in diagnosis of VCFs, although it could only provide limited detail about 3D anatomy structure or pathology of VCFs.

Deep learning (DL), a branch of machine learning, has already shown potential for assisting humans in various medical fields (3–6). A convolutional neural network(CNN) is a deep learning algorithm that is mainly designed to process image data and has grown to be a fundamental aspect of the medical field (7). In recent years, more and more radiomics model algorithms based on plain X-ray films have been developed in the wrist, humerus, hip, femur, shoulder, hand and foot (8–13). However, very few works are carried out using X-ray -based radiomics to predict VCFs. Recently, Chen et al have developed a DL–based model that distinguish fresh VCFs from digital radiography (DR) with sensitivity, specificity and AUC of 80%, 68% and 0.80 respectively. However, the clinical feasibility and benefit of DL–based model remain to be confirmed because no external and multicenter validation was performed in their study (14).

Thus, the aims of this study were to develop an X-ray-based deep learning model using a four-center dataset and determine whether the model could distinguish the type of VCFs and validate these findings in an independent external cohort.

## Materials and methods

### Datasets

This multicohort diagnostic study was performed with data from four hospitals in China. This study was approved by the institutional review board and ethics committee of the hospital (IRB 2022-108-01). Our retrospective study was approved by the institutional review board of the hospitals with a waiver for written informed consent. Patients in the prospective validation set of compression fractures provided written informed consent prior to participation.

For the development dataset, we evaluated the medical radiology reports of lumbar spine MRI in *Site 1* from 1 January 2014 to 31 October 2021 to determine acute, chronic, and pathological compression fractures. The inclusion criteria were as follows: (1) less than 2 weeks between Digital radiography (DR) and MRI examinations; and (2) the height of the vertebral body was reduced by at least 20% or 4 mm from the baseline height on the lateral radiography of the lumbar spine (15). The exclusion criteria were as follows: (1) surgical treatment for compression vertebral bodies such as internal fixation or bone cement filling; (2) lumbar spine presenting serious scoliosis or deformity; and (3) images with a low signal-to-noise ratio or foreign matter present.

**Figure 2:**
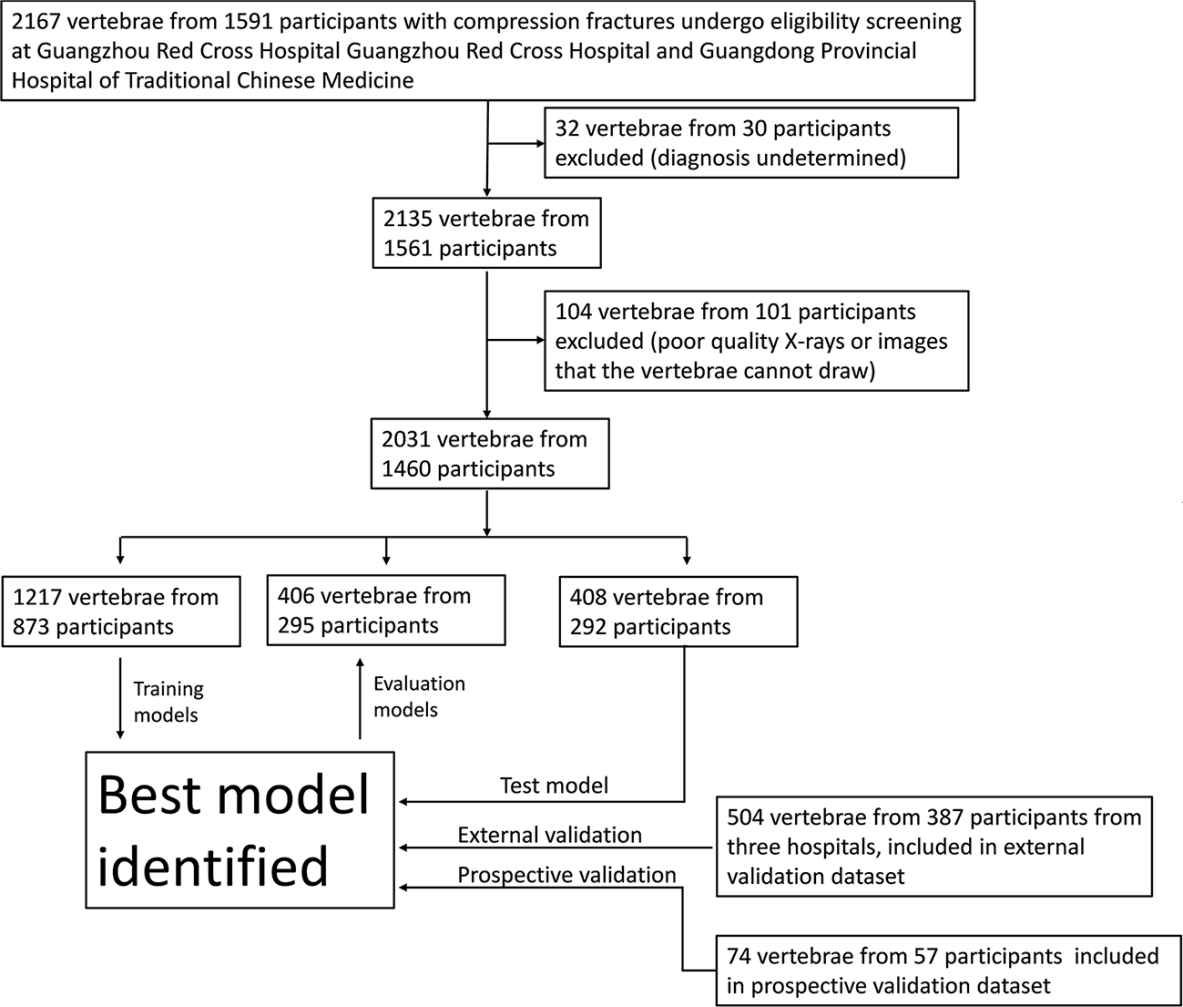
Workflow diagram for the development and evaluation of the deep learning model for compression fracture classification.

To verify the applicability of the classified diagnosis deep learning model in clinical practice, from October 1, 2019, to September 31, 2021, lumbar spine lateral X-ray images were also obtained from three hospitals across China: *Site 2*; *Site 3* and *Site 4*. In total, 2609 vertebrae from 1904 participants who underwent X-ray were enrolled at four independent hospitals (**Figure 2**).

From Nov 1, 2021, an independent dataset of consecutive participants undergoing lumbar spine X-ray in *Site 1* was prospectively enrolled. These participants were defined as the prospective validation set. In total, 74 vertebrae from 57 participants were enrolled at *Site 1* (**Figure 2**).

### Image reading and annotation

Delineated images of acute and chronic compression fractures are based on MRI diagnosis, and pathological compression fractures are diagnosed using MRI, positron emission tomography (PET) or histopathological results. When only MR images are available for a patient, at least two senior diagnosing physicians complete the diagnosis.

**Figure 3:**
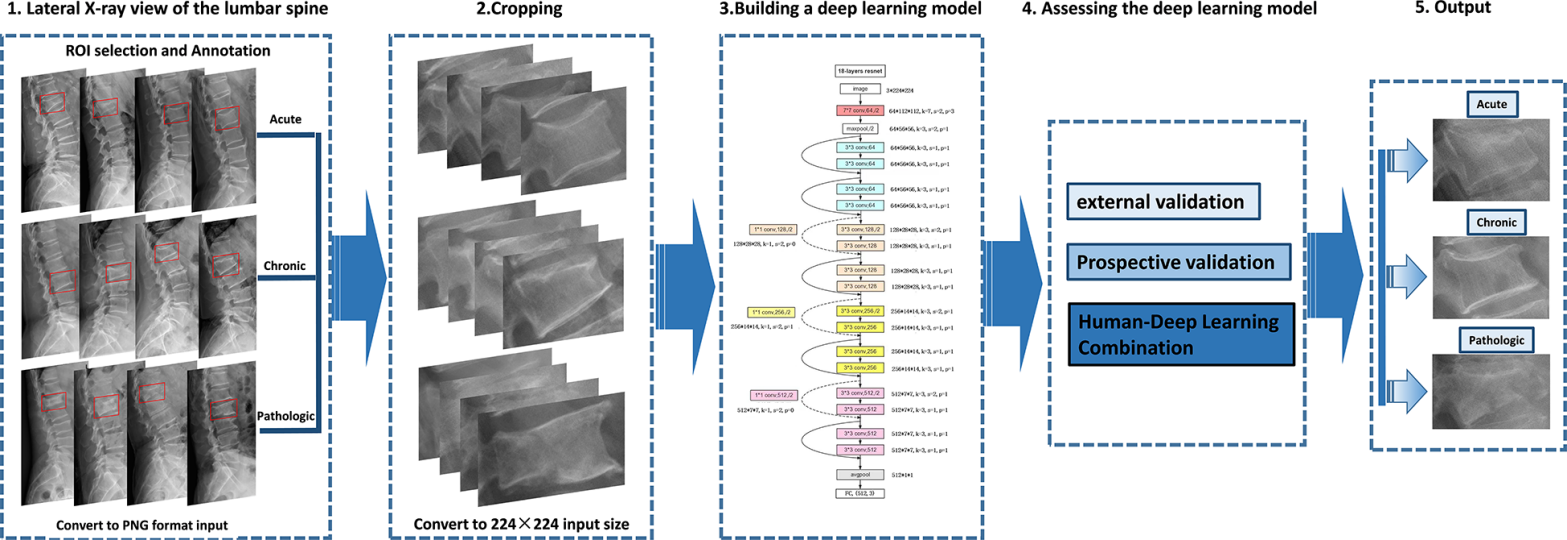
Deep learning architecture overview. First (step 1), compression fractures were reliably delineated and annotated on radiographs using labelImg software. For the step, the radiograph was converted to PNG format. Then (step 2), the cropped lateral X-ray of the lumbar spine was resized to 224×224 pixels and used as the input for a deep learning model. The third step (step 3) is to build a compression fracture classification model based on the Resnet-18 algorithm. Model performance was evaluated using external and prospective data and further validated using a radiologist-deep learning combination (step 4). As a result, we can provide adjunctive evaluation of lumbar compression fractures (acute, chronic, and pathological) (step 5).

Lesion regions of interest (ROIs) were manually represented with bounding boxes on lateral radiographs of the lumbar spine by an experienced radiologist using the LabelImg software (https://pypi.org/project/labelImg/) and annotated (224×224) (**Figure 3**).

### Model training

The images from the development dataset were randomly assigned with a ratio of 8:2 for the training datasets (the deep learning model for compression fracture classification) and the testing datasets (for evaluating the performance of the deep learning model). The image of the training set was enhanced, using horizontal flips, vertical flips, and rotations at random angles.

**Figure 4:**
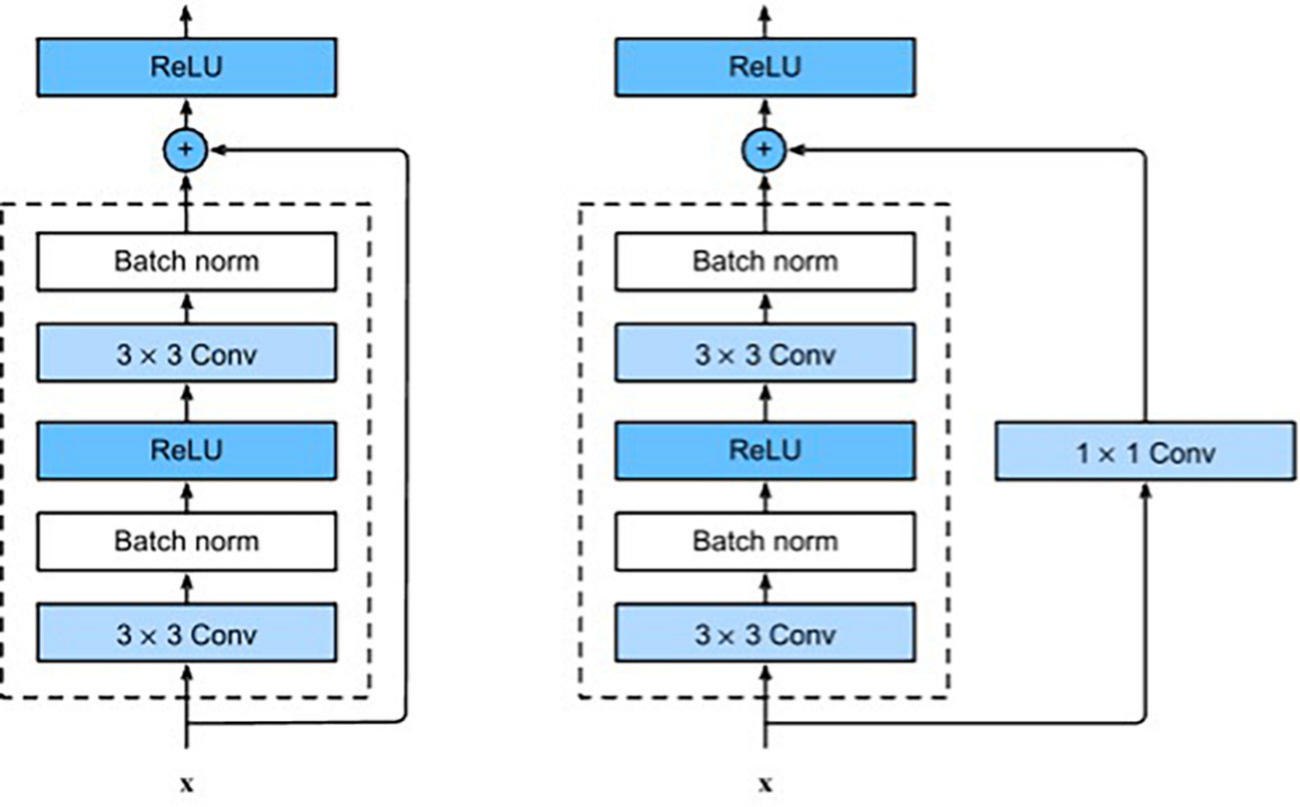
ResNet block with and without 1×1 onvolution, which transforms the input into the desired shape for the addition operation.

ResNet is a type of CNN where the input from the previous layer is added to the output of the current layer. This skip connection makes it easier for the network to learn and brings better performance. The ResNet architecture has been successful in many tasks, including image classification, objection detection, and semantic segmentation. In addition, because ResNet is composed of layers, these networks can obtain any level of spatial representation at any depth. Each ResNet block has 2 convolutional layers (excluding the 1×1 onvolutional layer), and we connect these two residual blocks as a module. We use 4 such modules, so there are a total of 16 convolutional layers (**Figure 4**). Together with the first 7×7 onvolutional layer and the final fully connected layer, there are 18 convolutional layers in total, which is ResNet-18 (16).

In this study, we used the ResNet-18 architecture model, and the input image was resized to 224 × 224 pixels and was normalized with mean= [0.485,0.456,0.406] and std= [0.229,0.224,0.225]. We then fine-tuned the model using a dataset of lateral lumbar spine radiographs of acute, chronic, and pathological compression fractures. Codes are available at https://github.com/Xiongyuchao/VCFNet.

### Validation of the model

We first validated the performance of the model in classifying VCFs using the testing dataset. We then evaluated the robustness of this model using an external validation dataset from three participating hospitals. Finally, the model was evaluated using prospective data including 74 vertebrae of 57 patients from *Site 1*.

### Validation of the radiologists’ visual diagnoses combined with DL-model based on collaboration dataset

In addition to the classification research of independent observers, the collaborative research of human and deep learning was also carried out to simulate a real clinical setting. We randomly selected 30% of the images from all external validation sets as the “collaboration dataset”. Three radiologists with different levels of expertise (trainee, competent, and expert) were asked to independently complete the same test images and compare their results with those of the model. Then the three radiologists reevaluated all the same test images independently after knowing the DL-model diagnosis. These radiologists were not involved in the selection and labeling of images, and the images were obfuscated and unidentified prior to evaluation. The expert radiologist was a professor with more than 20 years of experience in musculoskeletal diagnosis. The competent radiologist was a radiologist with 7 years of experience and completed standardized training for practicing physicians. The trainee is a radiologist with 2 years of experience and obtained the qualification certificate of a licensed doctor.

### Statistical analysis

All computer codes used for data analysis are stored in GitHub(https://github.com/Xiongyuchao/VCFNet). We used receiver operating characteristic (ROC) curves to demonstrate the ability of deep learning algorithms to classify VCFs. An ROC curve is generated by plotting the ratio of true positive cases (sensitivity) to false positive cases (1-specificity) by varying the predicted probability threshold. A larger area under the ROC curve (AUC) indicates better diagnostic performance.

## Results

### VCF and clinical datasets

**Table 1** provides an overview of the participant characteristics and the VCF classification data. 1003 vertebrae of acute compression fractures, 861 vertebrae of chronic compression fractures, and 167 vertebrae of pathologic vertebrae were included in the development dataset. In addition, 504 vertebrae from 387 participants were used to test the deep learning classification model at three external participating hospitals, and 74 vertebrae from 57 participants were prospectively collected at *Site 1* for the prospective validation dataset (**Figure 2**).

**Table 1:** Patient characteristics.

	Development Dataset		External validation			Prospective dataset	P value
	Training dataset	Testing dataset	GZFPH dataset	WHTH dataset	HB672H dataset		
No. of vertebral readings	1623	408	111	217	176	74	
No. of Patients	1168	292	97	147	143	57	
Age (years)	72.93±13.56	74.10±12.82	71.86±12.29	71.41±15.31	71.98±13.87	73.84±15.87	P<0.05
Sex							P<0.05
Male	365	92	32	65	61	13	
Female	803	200	65	82	83	44	
Compression fracture classification							
Acute	802	201	62	113	94	38	
Chronic	688	173	40	95	57	31	
Pathologic	133	34	9	9	25	5	

GZFPH, Guangzhou First People’s Hospital; WHTH, Wuhan Third Hospital; HB672H, Hubei 672 Integrated Traditional Chinese and Western Medicine Orthopaedic Hospital.

### Establishment of deep learning model

After 25 epochs, the training procedure was ended, with no further improvement in accuracy and cross-entropy loss on training, and testing. An accuracy of up to 95.9% was observed in the training set and 77.7% in the testing set (**Appendix**).

### Deep learning model performance on the VCF test set

**Figure 5:**
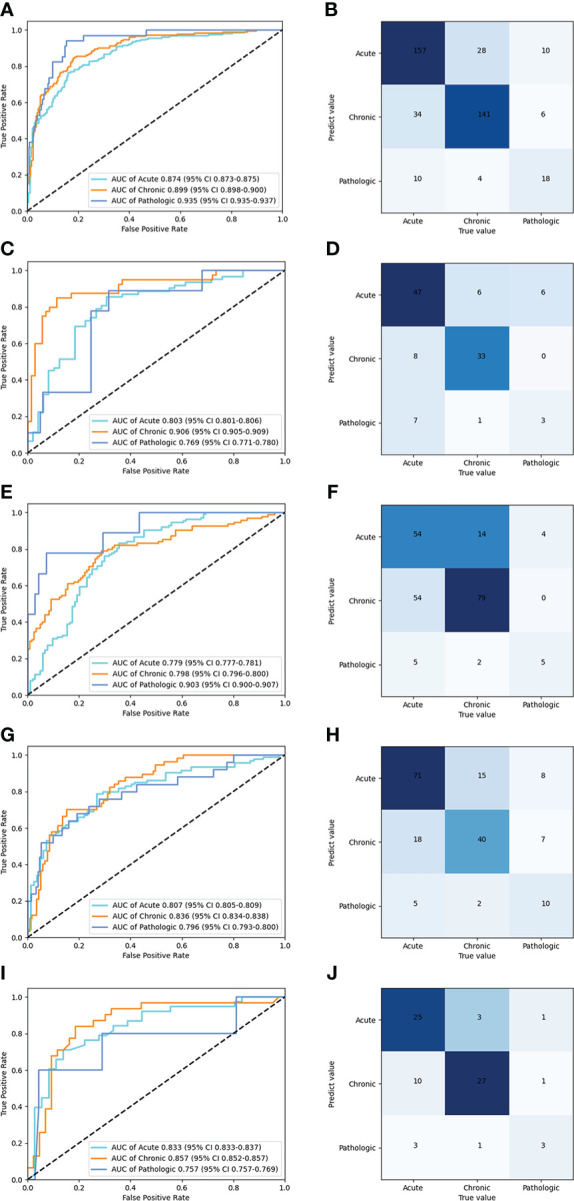
Performance of the deep learning model in the classification of acute, chronic, and pathologic compression fracture in X-ray images, in the internal and external validation datasets. ROC **(A)** and normalized confusion matrices **(B)** of the classification mode in the testing dataset. ROC **(C)** and normalized confusion matrices **(D)** of the classification mode in the GZFPH dataset. ROC **(E)** and normalized confusion matrices **(F)** of the classification mode in WHTH dataset. ROC **(G)** and normalized confusion matrices **(H)** of the classification mode in HB672H dataset. ROC **(I)** and normalized confusion matrices **(J)** of the prospective dateset.

**Table 2** shows the performance of the deep learning model in the classification of compression fractures in all five testing sets (**Figure 5**). Classification accuracies were 0.850 for the testing dataset and 0.829 for the prospective validation dataset. Classification accuracies for the external validation were 0.832 for *Site 2*, 0.757 for *Site 3*, and 0.792 for *Site 4*. Using the proposed model to assess the ability of each compression classification, the AUCs in acute, chronic, and pathological compression fractures were as follows: 0.874 (95% CI: 0.873, 0.875), 0.899 (95% CI: 0.898, 0.900) and 0.935 (95% CI: 0.935, 0.937) in the testing dataset, respectively; 0.803 (95% CI: 0.801, 0.806), 0.906 (95% CI: 0.905, 0.909) and 0.769 (95% CI: 0.771, 0.780) in the GZFPH dataset, respectively; 0.779 (95% CI: 0.777, 0.781), 0.798 (95% CI: 0.796, 0.800) and 0.903 (95% CI: 0.900, 0.907) in the WHTH dataset; 0.807 (95% CI: 0.805, 0.809), 0.836 (95% CI: 0.834, 0.838) and 0.796 (95%CI: 0.793, 0.800) in the HB672H dataset, respectively.

**Table 2:** Performance of the deep learning in different validation sets.

Dataset	AUC	Accuracy (%)	Sensitivity (%)	Specificity (%)	Precision (%)	F1 Score (%)	PPV (%)	NPV (%)
Testing dataset		85.0	77.5	88.7	77.4	77.4	77.5	88.7
Acute	0.874	79.9	78.1	81.6	80.5	79.3	80.5	79.3
Chronic	0.899	82.4	81.5	83.0	77.9	79.7	77.9	86.0
Pathologic	0.935	92.6	52.9	96.3	56.3	54.5	56.3	95.7
GZFPH dataset		83.2	74.8	87.4	75.7	75.2	74.8	87.4
Acute	0.803	75.7	75.8	75.5	79.7	77.7	79.7	71.2
Chronic	0.906	86.5	82.5	88.7	80.5	81.5	80.5	90.0
Pathologic	0.769	87.4	33.3	92.2	27.3	30.0	27.3	94.0
WHTH dataset		75.7	63.6	81.8	66.8	65.2	63.6	81.8
Acute	0.779	64.5	47.8	82.7	75.0	58.4	75.0	59.3
Chronic	0.798	67.7	83.2	55.7	59.4	69.3	59.4	81.0
Pathologic	0.903	94.9	55.6	96.6	41.7	47.6	41.7	98.0
HB672H dataset		79.2	68.8	84.4	68.6	68.7	68.9	84.4
Acute	0.807	73.9	75.5	72.0	75.5	75.5	75.5	72.0
Chronic	0.836	76.1	70.2	79.0	61.5	65.6	61.5	84.7
Pathologic	0.796	87.5	40.0	95.4	58.8	47.6	58.8	90.6
Prospective dataset		82.9	74.3	87.2	76.9	75.6	74.3	87.2
Acute	0.833	77.0	65.8	88.9	86.2	74.6	86.2	71.1
Chronic	0.857	79.7	87.1	74.4	71.1	78.3	71.1	88.9
Pathologic	0.757	91.9	60.0	94.2	42.9	50.0	42.9	97.0

GZFPH, Guangzhou First People’s Hospital; WHTH, Wuhan Third Hospital; HB672H, Hubei 672 Integrated Traditional Chinese and Western Medicine Orthopaedic Hospital.

### Human and deep learning collaboration

**Figure 6:**
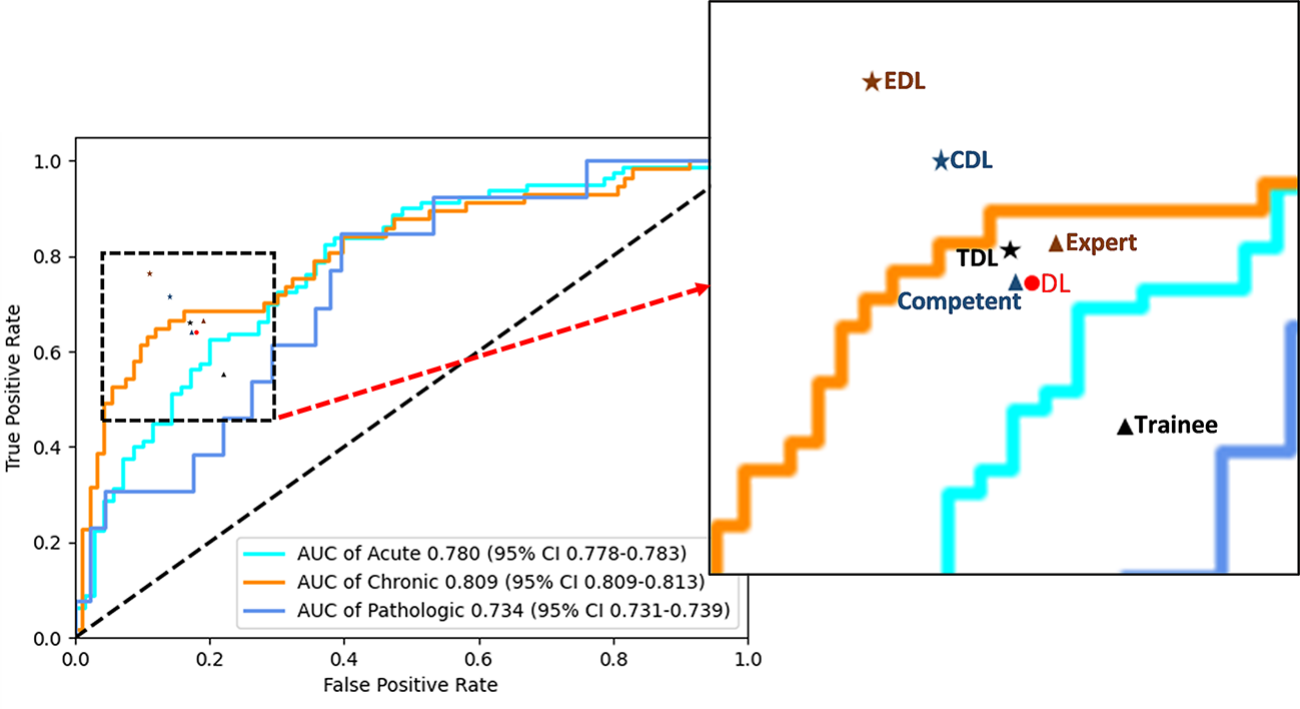
Performance of the deep learning and radiologists in classifying VCFs from X-ray images in the human and deep learning collaboration dataset. ROC of the classification mode in the human and deep learning collaboration dataset. The red dot, black triangle, black star, orange triangle, orange star, blue triangle, and blue star indicate the performance of deep learning (DL), trainee, trainee-deep learning collaboration (TDL), competent, competent-deep learning collaboration (CDL), expert, and expert-deep learning collaboration (EDL), respectively.

In the human and deep learning collaboration dataset, 80 vertebrae with acute compression fractures, 57 vertebrae with chronic compression fractures, and 13 vertebrae with pathologic compression fractures were included. The classification results of images from human and deep learning collaboration dataset by the deep learning and radiologists are shown in **Table 3**. The AUCs in acute, chronic, and pathological compression fractures were as follows: 0.780, 0.809, 0.734 for the deep learning model; 0.573, 0.618, 0.541 for the trainee radiologist; 0.701, 0.782, 0.665 for the competent radiologist; 0.707,0.732, 0.667 for the expert radiologist; 0.722, 0.744, 0.610 for the DL and trainee; 0.767, 0.779, 0.729 for the DL and competent; 0.801, 0.825, 0.751 for the DL and expert radiologist. The overall accuracy of the deep learning model was 0.764, which was significantly higher than that of the trainee radiologist (0.707), similar to the competent radiologist (0.769), and slightly lower than the expert radiologist (0.782). When combined with the deep learning model, the expert, competent, and trainee radiologists’ accuracy all increased significantly (0.853, 0.816, and 0.778, respectively). For sensitivity, combined with the deep learning model, the trainee, competent, and expert radiologists also significantly improved (0.560 *vs*. 0.667, 0.653 *vs*. 0.727, and 0.673 *vs*. 0.776, respectively). For the classification of pathological compression fractures, the sensitivity of expert radiologists was up to 0.385 and the deep learning model was only 0.308. However, when combined with the deep learning model, the expert radiologist, competent radiologist, and trainee radiologist all had increased sensitivity to pathological compression fracture (0.385 *vs*. 0.538, 0.462 *vs*. 0.538, and 0.154 *vs*. 0.308, respectively) (**Figure 6**). In addition, for pathological compression fractures, 6 out of 13 vertebrae were misjudged by all radiologists and the deep learning model.

**Table 3:** Performance of the deep learning versus radiologists in classifying compression fractures in the human and deep learning collaboration dataset.

Dataset	AUC	Accuracy (%)	Sensitivity (%)	Specificity (%)	Precision (%)	F1 Score (%)	PPV (%)	NPV (%)
Deep learning model		76.4	64.7	82.3	65.0	64.8	64.7	82.3
Acute	0.780	69.3	67.5	71.4	73.0	70.1	73.0	65.8
Chronic	0.809	71.3	68.4	73.1	60.9	64.5	60.9	79.1
Pathologic	0.734	88.7	30.8	94.2	33.3	32.0	33.3	93.5
Trainee radiologist		70.7	56.0	78.0	55.7	55.8	56.0	78.0
Acute	0.573	62.0	65.0	58.6	64.2	64.6	64.2	59.4
Chronic	0.618	64.0	52.6	71.0	52.6	52.6	52.6	71.0
Pathologic	0.541	86.0	15.4	92.7	16.7	16.0	16.7	92.0
Competent radiologist		76.9	65.3	82.7	69.9	67.5	65.3	82.7
Acute	0.701	69.3	58.8	81.4	78.3	67.1	78.3	63.3
Chronic	0.782	78.0	78.9	77.4	68.2	73.2	68.2	85.7
Pathologic	0.665	83.3	46.2	86.9	25.0	32.4	25.0	94.4
Expert radiologist		78.2	67.3	83.7	67.4	67.4	67.3	83.7
Acute	0.707	70.7	70.0	71.4	73.7	71.8	73.7	67.6
Chronic	0.732	74.0	70.2	76.3	64.5	67.2	64.5	80.7
Pathologic	0.667	90.0	38.5	94.9	41.7	40.0	41.7	94.2
Deep learning and trainee		77.8	66.7	83.3	67.9	67.3	66.7	83.3
Acute	0.722	72.0	70.0	74.3	75.7	72.7	75.7	68.4
Chronic	0.744	75.3	70.2	78.5	66.7	68.4	66.7	81.1
Pathologic	0.610	86.0	30.8	91.2	25.0	27.6	25.0	93.3
Deep learning and competent radiologist		81.6	72.7	86.0	73.4	73.1	72.2	86.3
Acute	0.767	76.7	76.3	77.1	79.2	77.7	79.2	74.0
Chronic	0.779	79.3	71.9	83.9	73.2	72.6	73.2	83.0
Pathologic	0.729	88.7	53.8	92.0	38.9	45.2	38.9	95.5
Deep learning and expert radiologist		85.3	77.6	89.1	77.4	77.5	77.6	89.1
Acute	0.801	80.0	80.5	79.5	80.5	80.5	80.5	79.5
Chronic	0.825	83.3	78.9	86.0	77.6	78.3	77.6	87.0
Pathologic	0.751	92.7	53.8	96.4	58.3	56.0	58.3	95.7

## Discussion

In this multicenter study, we successfully developed a classification model for acute, chronic and pathological compression fractures by using deep learning neural networks. The model demonstrated high accuracy and specificity in classifying compression fractures in retrospectively stored images as well as in a prospective observational setting. Furthermore, the diagnostic efficiency of deep learning model is higher than that of the trainee radiologists, similar to the competent radiologist, and slightly lower than the expert radiologist. The deep learning model combined with all three expertise levels of radiologists significantly improved the accuracy, specificity, and sensitivity of evaluating compression fractures. To the best of our knowledge, this is the first multicenter study to apply deep learning with CNNs to the classification of acute, chronic, and pathological compression fractures.

Previous studies have used information from radiographs to classify compression fractures. Usually, plain radiographs are initially performed to diagnose acute compression fractures by observing small changes such as endplate rupture and anterior wall protrusion (17) and diagnosis of pathological compression by cortical penetration, trabecular bone destruction, vertebral bone density, and special compression morphology. Although studies of these conventional features have provided guidance for the classification of compression fractures, the information provided by lumbar spine X-rays is limited, and compression fractures can only be simply assessed from morphological and partial imaging signs. The diagnosis of compression fractures, which is subjective and largely depends on the skills and experience of the diagnosing physician, needs to be based on professional knowledge and the accumulation of diagnostic experience to improve the accuracy of diagnosis. CT (18, 19), MRI (19–22), and PET (23) have shown great advantages in the classification of compression fractures, but their clinical applicability has been limited because of patient noncooperation, high costs, and the need for specialized training in tomographic image interpretation. The proposed deep learning CNN has instead been found to provide auxiliary diagnosis to non-professional radiologists to improve performance (competent from 0.756 to 0.816 and trainee from 0.707 to 0.796) on compression fracture classification, both exceeded the expert level (0.764). The deep learning model demonstrated high accuracy and specificity in classifying compression fractures in retrospectively stored images as well as in a prospective observational setting. Furthermore, the diagnostic efficiency of deep learning model is higher than that of the trainee radiologists, similar to the competent radiologist, and slightly lower than the expert radiologist (**Figure 6**). The deep learning model combined with all three expertise levels of radiologists significantly improved the accuracy, specificity, and sensitivity of evaluating compression fractures. Thus, for developing countries such as China or countries with scarce medical resources, where there is an unequal distribution of urban and rural medical resources, this deep learning CNN can help bridge the classification of compression fractures between national and primary hospitals. In addition, with deep learning model assistance, the classification accuracy of three radiologists with different levels of experience was also improved significantly.

The classification sensitivity of pathological compression fractures was found to be low for all three radiologists and the deep learning model. Even the combination of all three radiologists and the deep learning model was still low. In addition, 6 vertebrae with pathological compression fractures were misdiagnosed by all three radiologists and the deep learning model. We speculate that the main reasons for these false negatives are the low contrast of X-ray images, the fact that there were no obvious signs of bone damage on the vertebral body, intestinal gas interference and bilateral shadows of the vertebral body, which may be an insurmountable limitation caused by the principle of X-ray imaging. However, deep learning model-assisted diagnosis can improve the sensitivity of pathological compression fracture classification, albeit still at a low level. Furthermore, given the high accuracy of classification of acute and chronic compression fractures, deep learning could be considered cost-effective.

There are few studies on compression fractures using deep learning methods, especially based on conventional X-ray images. Only one study used deep learning to evaluate acute and chronic compression fractures on radiographs, which included 1099 patients and used image data from anteroposterior and lateral lumbar spine radiographs as input to a neural network. This study achieved a sensitivity, specificity and AUC of 0.80, 0.68, and 0.80, respectively (14). The clinical applicability of CNN models may be limited as a result of dichotomous disease surveys and retrospective and single-institution studies in homogeneous hospitals. By comparison, the deep learning model of this study demonstrated an overall high accuracy in classifying compression fractures in three retrospective validation sets, suggesting that the model may be generalizable across a variety of scenarios.

Despite these remarkable results, our study has some limitations. First, the subjects who received internal fixation or cementation or who presented with severe scoliosis/deformity were excluded in the development dataset, which may lead to bias. Second, from the nature of CNNs, since a neural network only provides a classification of an image and associated compression fractures, there is no explicit feature definition. Third, the ROIs of the compression fracture were delineated by a manual rectangle. The training of the model relies on the accurate identification of compressed vertebral bodies by radiologists, which requires manual delineation by radiologists. Although there are some differences in the ROIs drawn by different doctors, they will be uniformly processed and then classified after the images are imported into the model. Currently ROI delineation could be finished using automatic, semi-automatic and hand-crafted methods. However, automatic and semi-automatic methods may have a certain deviation which need to be further adjusted manually. Fourth, due to the small numbers of pathological compression fractures in this study with low sensitivity, it is likely the features of pathological fracture might be different from the osteoporotic/traumatic compression fracture and will require a large and specific pathological database to clarify the utilization of deep learning model assistance. Therefore, more studies with larger numbers of patients are required to provide stronger evidence for the accuracy of deep learning models in the prediction of pathological compression fractures. Finally, deep learning model alone was not adequate to detect pathological compression fracture due to the absence of clinical information. Thus, the general applicability of our results in clinical practice could be affected. Clinical information is required in future study to validate the performance of deep learning models.

In conclusion, a multiclass deep learning model for compression fractures on radiographs was developed and validated. The classification performance of the model surpassed that of trainee radiologists and was comparable to that of experienced radiologists. When the skills of the radiologists were combined with a deep learning model, better diagnostic performance was observed, which could improve the accuracy of diagnostic classification, thereby improving the diagnostic workflow for patients with compression fractures. We expect to establish an artificial intelligence-assisted diagnosis platform for compression fracture based on X-ray images to provide patients and clinicians with free access to telemedicine assistance, aiming to eliminate the diagnosis and treatment gap between national hospitals and grassroots hospitals.

## Data availability statement

The excel files containing raw data included in the main figures and tables can be found in supplementary material. The lumbar spine X-ray imaging data and clinical information are not publicly available for patient privacy reasons but are available from the corresponding authors upon reasonable request (FX, YCX and XWZ). The models and the code used to test and evaluate the model is available on GitHub (https://github.com/Xiongyuchao/VCFNet). The remaining data are included in the article/**Supplementary Material**, further inquiries can be directed to the corresponding author/s.

## Ethics statement

The studies involving human participants were reviewed and approved by the institutional review board and ethics committee of the Guangzhou Red Cross Hospital (IRB 2022-108-01). The patients/participants provided their written informed consent to participate in this study.

## Author contributions

Conception and design: FX, YCX; acquisition of additional data: FX, YCX, GXY, YYL, QPD, WYJ, DLW, SC; analysis and interpretation of data: FX, YCX, GXY, ZPL, XWZ; statistical analysis: FX, YYL, GXY; drafting of the manuscript: FX, YCX; critical revision of the manuscript: FX, XWZ; study supervision: XWZ. All authors listed have contributed substantially to the design, data collection and analysis, and editing of the manuscript.

## Supplementary material

The models and the code used to test and evaluate the model is available on GitHub (https://github.com/Xiongyuchao/VCFNet).
